# Electron Temperature Measurements Using a Two-Filter Soft X-ray Array in VEST

**DOI:** 10.3390/s23208357

**Published:** 2023-10-10

**Authors:** M. W. Lee, S. Lim, W. Jeong, S. Kim, J. H. Kim, Y. S. Hwang, C. Sung

**Affiliations:** 1 Department of Nuclear and Quantum Engineering, Korea Advanced Institute of Science and Technology, Daejeon 34141, Republic of Korea; mwlee@kaist.ac.kr; 2 Department of Energy Systems Engineering, Seoul National University, Seoul 08826, Republic of Korea; lsb9012@snu.ac.kr (S.L.); wjeong1227@snu.ac.kr (W.J.); sungfe90@snu.ac.kr (S.K.); rlawnd0627@snu.ac.kr (J.H.K.); yhwang@snu.ac.kr (Y.S.H.)

**Keywords:** soft X-ray, two-filter method, electron temperature, bremsstrahlung, AXUV

## Abstract

A multichannel soft X-ray (SXR) array has been developed to measure the electron temperature in the Versatile Experiment Spherical Torus (VEST). To estimate electron temperature using the two-filter method applied to SXR intensity, we designed a pinhole camera that has two photodiode arrays with different metallic filters. We also adopted a filter wheel and tested various filter parameters to find the optimal filter set. Through tests, the combination of aluminum and beryllium was found to be the most suitable for the current experimental conditions in VEST. The filtered SXR signals were acquired with a low-noise preamplifier, exhibiting sufficient signal-to-noise ratios for electron temperature estimation based on the intensity ratio of two signals obtained with different filters. The estimated electron temperature from the developed two-filter SXR array showed reasonably matched levels and consistent trends with Thomson scattering measurements. Error contribution from impurity line emission is also discussed.

## 1. Introduction

Information on electron temperature (*T_e_*) evolution with high temporal resolution is of prime importance in fusion research, as it provides critical insights into plasma behavior such as magnetohydrodynamics (MHD) instabilities [1,2,3,4,5]. In the Versatile Experiment Spherical Torus (VEST) [6], a Thomson scattering diagnostic and triple probe have been utilized to measure local *T_e_* profiles. The Thomson scattering diagnostic measures five locations simultaneously but only every 1 ms [7], and the triple probe covers only edge plasmas, not the core region. These two diagnostics are therefore not sufficient to obtain radial *T_e_* profiles with high temporal resolution. This motivated us to develop a soft X-ray (SXR)-based *T_e_* diagnostic for complementary use. Among the various *T_e_* measurement techniques, the use of SXR radiation offers several advantages, particularly high temporal resolution (~MHz) and wide measurement regions through multichannel measurements that can cover the whole plasma. 

For *T_e_* estimation using soft X-rays, there are two representative methods: pulse height analysis (PHA) and the two-filter method. The PHA measures the energy spectrum of soft X-rays that pass through a single X-ray filter to estimate *T_e_*. In previous studies, silicon drift detectors (SDD) are commonly used for applying PHA to several fusion devices [8,9,10]. However, due to energy resolution (~180 eV) [11] and low detection efficiency of SDD below 1 keV [12], applying PHA to sub-keV range is challenging. Therefore, in this work, a new VEST SXR array was designed and installed that applies the two-filter method [13,14,15,16,17] to estimate *T_e_* from the measured bremsstrahlung emission. To measure soft X-rays, multichannel absolute extreme ultraviolet (AXUV) photodiodes with sensitivity at less than 1 keV are used in this study. With the multichannel measurements, *T_e_* profiles can be inferred directly from the ratio of the intensity levels of the SXR signals measured from the identical line-of-sight (LOS) but through different filters. We designed the system to measure temperatures around 10–200 eV for VEST, suggested by previous measurements using the existing Thomson scattering system and triple probe. For such a relatively low-temperature application, the choice of filter sets is particularly important because the narrow energy region should be resolved while the filter materials are limited at low energies. Moreover, a sufficient signal-to-noise ratio (SNR) is also required to obtain the ratio of the two signals. In this work, we successfully designed and installed a proper two-filter SXR array for VEST application through simulation and experiment.

This paper describes the development and initial results of the new two-filter SXR array in VEST. Section 2 presents the design of the diagnostic system, including the filter selection process for the optimal filter set for VEST. Experimental SXR measurements during VEST operation are discussed in Section 3, and initial *T_e_* estimations are presented in Section 4 with a consideration of impurity line emission as an error contribution. Finally, Section 5 summarizes the results with future upgrade plans.

## 2. Design of the Two-Filter Soft X-ray Array

### 2.1. Selection of Soft X-ray Filters

The two-filter method estimates *T_e_* by comparing the SXR intensity levels through two different filters on the same LOS crossing the plasma. To select proper filters, we first calculated the expected *T_e_* that will later be estimated from the ratio of two SXR signals using different filters. The SXR emissivity from the bremsstrahlung continuum of the plasma is a convolution of plasma parameters such as effective charge (*Z_eff_* = $\sum_{j}Z_{j}^{2}n_{j}/Z_{j}n_{j}$, *j* is ion species), electron density (*n_e_*), and *T_e_*. Therefore, the emissivity, *P_rad_*, through a certain filter with its transmittance, eff(*E*), is given by [18]

(1) $$P_{rad}=C\cdot \int \frac{n_{e}^{2}Z_{eff}}{\sqrt{T_{e}}}e^{-\frac{E}{T_{e}}}\mathrm{eff}\left(E\right)dE.$$

Here, the constant *C* includes the Gaunt factor and geometrical factors, and *E* is the photon energy. Equation (1) can be rewritten to highlight its dependency on *T_e_* as

(2) $$P_{rad}=C^{*}\cdot \int \frac{1}{\sqrt{T_{e}}}e^{-\frac{E}{T_{e}}}\mathrm{eff}\left(E\right)dE,$$

where $C^{*}=Cn_{e}^{2}Z_{eff}$. Since *Z_eff_* and *n_e_* are not affected by photon energy *E*, these two factors are included in $C^{*}$.

By dividing the two signals with two different filters, the constant term cancels out, and we can obtain a function only depending on *T_e_*:

(3) $$R\left(T_{e}\right)=\frac{\int e^{-\frac{E}{T_{e}}}eff_{1}\left(E\right)dE}{\int e^{-\frac{E}{T_{e}}}eff_{2}\left(E\right)dE}$$

**Figure 1:**
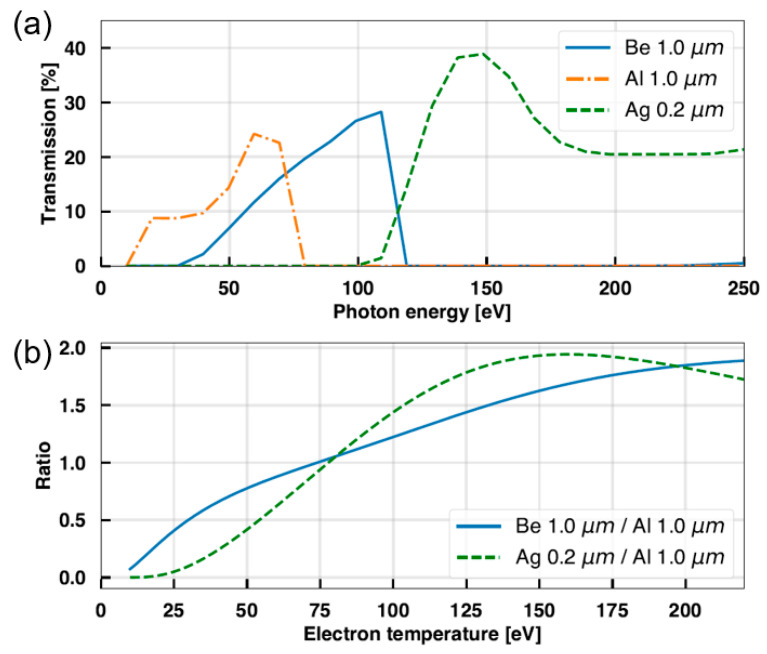
(**a**) Transmission of Be 1.0 μm, Ag 0.2 μm, and Al 1.0 μm filters. (**b**) Ratio–temperature curves of two filter sets.

Equation (3) should be a monotonic function to obtain *T_e_* by measuring the ratio of SXR intensity levels with two different filters. Since the shape of the ratio–temperature curve (*R*(*T_e_*) curve) obtained from Equation (3) varies with the filter combination, it is important to select a proper filter set for the target *T_e_* range. Considering the measured *T_e_* of VEST Ohmic discharge (10–200 eV) using previous diagnostics, we selected aluminum (Al) 1.0 μm, beryllium (Be) 1.0 μm, and silver (Ag) 0.2 μm for the initial filter sets. Figure 1a shows the transmission curves of the individual filters, and *R*(*T_e_*) curves, calculated via Equation (3) and the transmission curves, are plotted in Figure 1b. Among the three filters, only Al can transmit the low-energy photons from 10 eV, so we chose Al as one reference filter to obtain the intensity ratio. To measure higher energy photons around 100 eV, Be or Ag could be a candidate. As shown in Figure 1b, the Ag/Al combination shows a stiffer curve compared to Be/Al, which means a higher sensitivity in *T_e_* estimation. However, the maximum measurement range with Ag/Al is only up to 150 eV, which is lower than that of the Be/Al combination (10–200 eV).

### 2.2. Pinhole Camera

**Figure 2:**
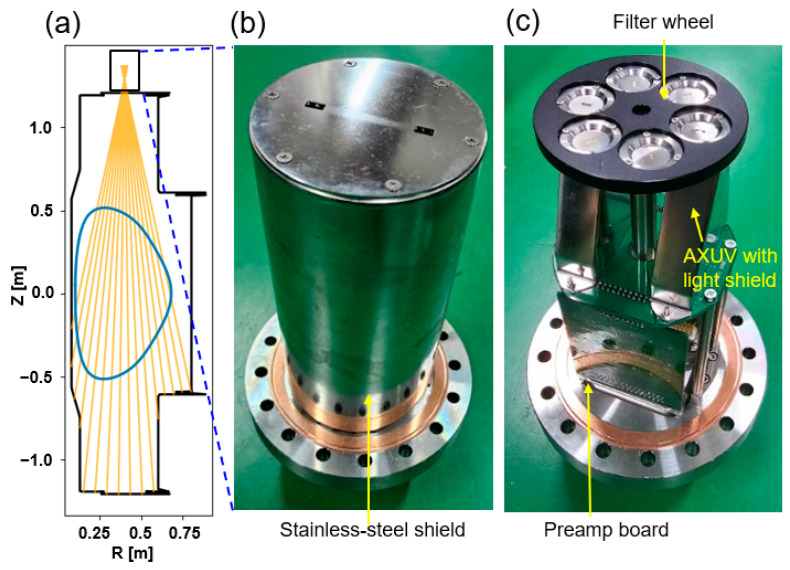
(**a**) Poloidal LOSs of the SXR array installed at the VEST top port. (**b**) Complete pinhole camera with a stainless-steel shield. (**c**) Interior of the camera. A filter wheel and light covers were installed along the light path, and the preamp electronics were directly connected to absolute extreme ultraviolet (AXUV) photodiodes and a D-sub vacuum feedthrough.

A pinhole camera capable of mounting multiple filters was designed to measure SXR. The camera was installed at the VEST top port to cover the entire plasma with 16 LOSs in a poloidal plane, as shown in Figure 2a. A picture of the camera with a stainless-steel shield is shown in Figure 2b. Two pinholes were placed in the toroidal direction with 4 cm spacing. Considering the major radius of typical VEST plasmas of ~0.4 m, 4 cm toroidal spacing corresponds to ~6 degrees in terms of toroidal angle, which is quite a small separation in the toroidal direction. Therefore, we suppose that the two parallel pinholes measure almost the same poloidal plane.

Figure 2c shows the interior of the camera. In the front of the camera, a filter wheel was attached to the shaft at the camera center. The shaft was then connected to the outside of the vacuum through a feedthrough. This shaft can be rotated from the outside. We adopted the filter wheel to test various parameters such as filter material, thickness, and pinhole size.

**Figure 3:**
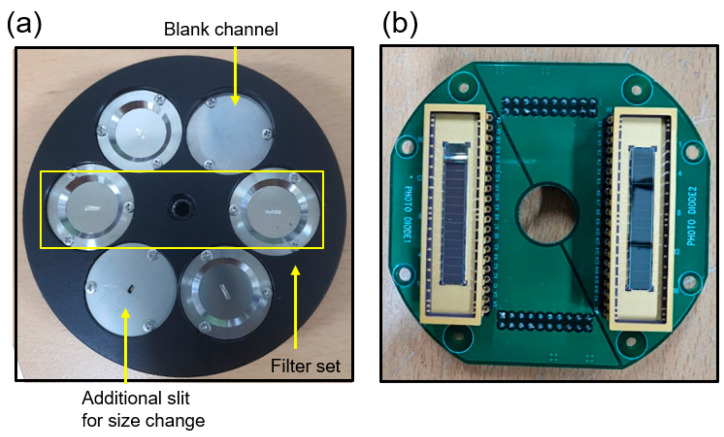
(**a**) Filter wheel that can load six different filters. Two filters placed across the center correspond to a filter set. (**b**) Detector printed circuit board to load two 16-channel AXUV photodiodes behind the filter wheel.

**Figure 4:**
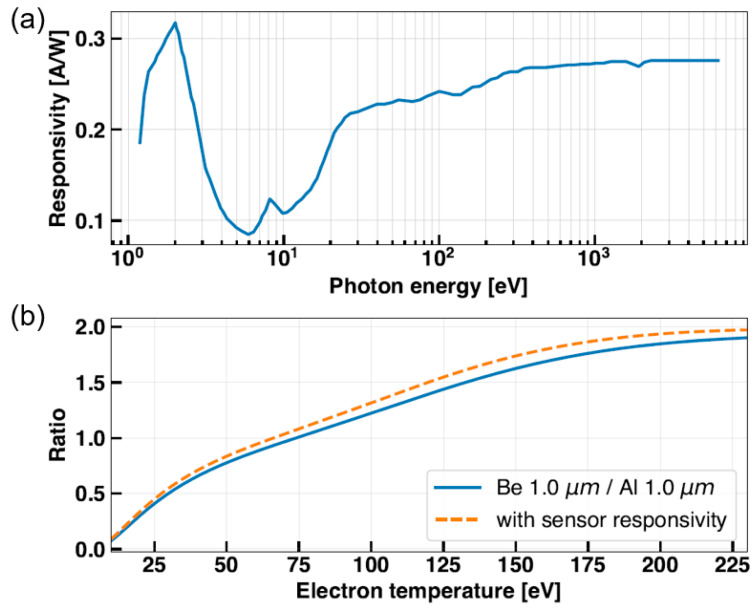
(**a**) Responsivity of an AXUV photodiode to photon energy. (**b**) Changes in the ratio–temperature curve with consideration of sensor responsivity.

As shown in Figure 3a, the filter wheel can load six filters, corresponding to three filter combinations for the two-filter method. To prevent tearing by minimizing the exposed area, thin metallic filters were attached to just cover the pinholes (slits) of size 1 × 4 mm^2^. The effective size of the slits could be changed for the tests by placing additional slits. A blank channel was also made to check for unexpected stray light signals. We used two 16-channel AXUV photodiodes as SXR detectors. The two detectors were mounted on a detector printed circuit board (PCB) behind the filter wheel, as depicted in Figure 3b. The slits loaded on the filter wheel and the detectors were separated by a focal length of 6 cm, and a trapezoidal light shield was positioned between them to block stray light. It is noteworthy that AXUV photodiodes have different sensitivities depending on the incident photon energy [19]. Figure 4a shows the responsivity of an AXUV photodiode to photon energy. While the change in sensitivity can be treated as constant in the keV region, it was more distinct under 100 eV, which was our range of interest. Therefore, we also added a sensitivity function *S*(*E*) to the integrand of Equation (3) for precise estimation. Then, the revised equation becomes,

(4) $$R\left(T_{e}\right)=\frac{\int e^{-\frac{E}{T_{e}}}eff_{1}\left(E\right)S\left(E\right)dE}{\int e^{-\frac{E}{T_{e}}}eff_{2}\left(E\right)S\left(E\right)dE}.$$

Without the consideration of detector sensitivity, *T_e_* could be overestimated by about 10% in the VEST range, as shown in Figure 4b.

### 2.3. Electronics and Data Acquisition

**Figure 5:**
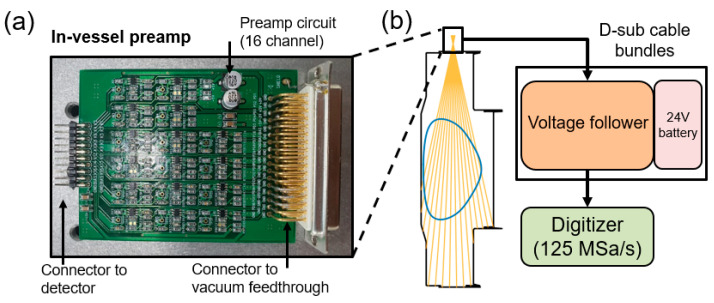
(**a**) Sixteen-channel preamp board installed inside the pinhole camera. (**b**) Block diagram of the data acquisition system from the inboard preamp to the digitizer. The voltage follower and preamp battery are located outside the vacuum vessel.

The SXR signals were first amplified at the preamp inside the pinhole camera and then went to a voltage follower as a buffer to further minimize the noise. Then, the signals were acquired at a digitizer, as shown in Figure 5b. The filtered SXR photons onto the AXUV photodiodes were expected to invoke a photocurrent on the order of nA, considering the VEST plasma parameters. Therefore, amplification with a high level of transimpedance gain was required for the measurements. In order to measure the small-amplitude SXR signals, a preamplifier was designed to convert the photocurrent measured from the photodiode into voltage with a gain of 10^6^ V/A. Figure 5a shows the customized preamplifier used for the measurements. The operational amplifier (ADA4851) employed in the preamplifier has a high-speed characteristic with a –3 dB bandwidth of 100 MHz, ensuring a sufficiently fast response time for instability measurements. To minimize additional noise that may arise from extended connections between the photodiode and amplification stages, the preamplifier PCB was positioned within the pinhole camera inside the vacuum vessel. Furthermore, the preamplifier was designed to be connected via short connectors between the PCBs, eliminating some cable connections. The preamplifier PCB was connected to the photodiode PCB through pin connectors, and the amplified signals were linked to the vacuum feedthrough through D-sub connectors. Through the D-sub cables, the signals were connected to the external preamp board (voltage followers) located outside the vacuum. To exclude additional noise from the power supply, a 24 V battery located outside the vacuum was employed for the preamp system.

The measured signals, after passing through the voltage followers, were finally transmitted to a multichannel digitizer and stored in a database. A digitizer capable of a maximum sampling rate of 125 MHz was utilized in the system (CAEN VX2740). Given that the instability signals observed in VEST typically have frequencies below 100 kHz, the measurements were conducted by adjusting the sampling rate to 1/128 (0.977 MHz) to reduce the data size.

## 3. Experimental SXR Measurements

**Figure 6:**
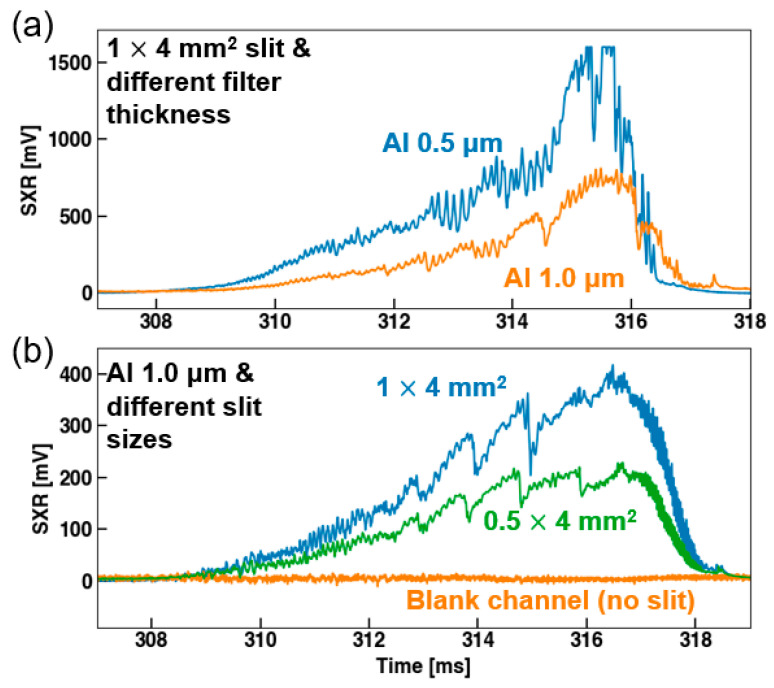
SXR signals with different filter settings. (**a**) Results of different Al filter thickness and fixed slit size. (**b**) Results of different slit size and fixed Al filter thickness (1.0 μm). The blank channel without a slit was tested to check for stray light.

We first observed the SXR signal amplitude while varying filter thickness and slit size to determine an appropriate measurement condition in which suitable signal levels can be obtained within the measurable range of the digitizer (1 V peak-to-peak). For this purpose, a 1.0 μm Al filter was used because it was expected to show the highest signal level among the filters by having transmission at the lowest energy. Figure 6 shows the test results of one measurement channel passing through the core region of the VEST plasma. The tests were performed by repeating similar discharges that had a plasma current (*I_p_*) around 100 kA. In Figure 6a, we changed the thickness of the Al filter while fixing the slit size. The Al 0.5 μm filter showed a signal level up to 1.7 V, which was almost twice that of the 1.0 μm filter. However, some signals were saturated over the digitizer range because there were some offset levels on each channel. The offset levels were from leakage currents of the diode channels and were up to 500 mV after amplification. For analysis, the offset levels were post-processed by subtracting the average level without the plasma signal.

We also varied the slit width in the poloidal direction by fixing the toroidal slit width to 4 mm. It was found that the signal level was enhanced as the slit width increased, but it remained within the measurable range of the digitizer, as shown in Figure 6b. We also observed that a signal level ranging from 100 mV (edge) to 700 mV (core) could be obtained when utilizing a 1 × 4 mm^2^ slit with a 1.0 μm Al filter. Considering the results shown in Figure 6 and the measurement range (0–2 V) with offset levels, we selected the Al 1.0 μm filter with the 1 × 4 mm^2^ slit, which showed a sufficient signal level for the intensity ratio comparison. In addition, an assessment was conducted to determine the effects of stray light on the measurements. To verify the presence of stray light signals, a blank cover, as shown in Figure 3a, was loaded onto the filter wheel to provide a blank channel without slits or filters. Any signal observed here would imply the presence of light leakage from structural gaps. As shown in Figure 6b, the blank channel did not exhibit any significant signal increase during plasma discharge. Hence, it was confirmed that the measured signals were only from the photons that passed through the filters.

**Figure 7:**
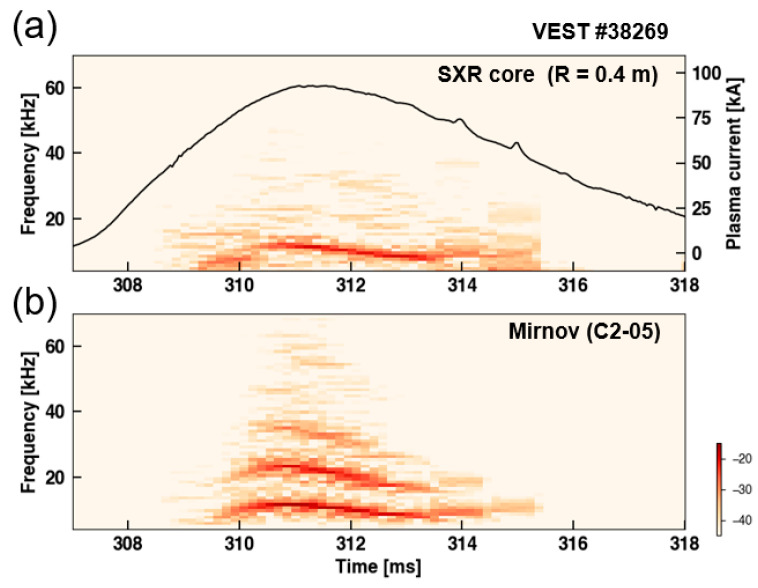
(**a**) Fourier spectrogram of a core SXR channel. The plasma current of the discharge is also plotted. (**b**) Mirnov coil signal near the toroidal location of the SXR array.

To examine the potential for identifying instabilities from the SXR signals, SXR signals were compared with Mirnov coil signals. Figure 7 plots the Fourier spectra of both SXR and Mirnov coil signals. The Mirnov coils in VEST [20] are located at four different toroidal positions on the outboard midplane. The Mirnov signal in Figure 7b was obtained from the toroidal position closest to where the SXR array was installed. From the Mirnov signal, a mode around 10 kHz with harmonic frequencies was observed. This mode could also be observed in the SXR array installed on the vertical port, as shown in Figure 7a. The observed consistency between SXR and magnetic coil signals supports the usefulness of this new system as a fluctuation diagnostic in VEST.

## 4. Initial Electron Temperature Estimation

### 4.1. Ratio Comparison of Be/Al Filter Sets

**Figure 8:**
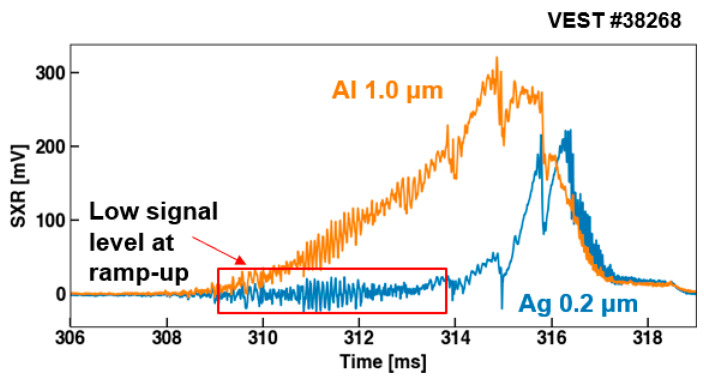
SXR signals from Al and Ag filters during Ohmic discharge. The Ag filter showed a quite low signal level during *I_p_* ramp-up.

In order to apply the two-filter method, the signal ratio from different filter combinations was evaluated. Subsequently, *T_e_* was estimated by interpolating the ratio onto the previously calculated *R*(*T_e_*) curve from Section 2.1. Besides the Al filter, Ag and Be filters were utilized in this test. Among these, Al demonstrated the highest signal intensity as it has transmission at the lowest energy. Therefore, the Al filter signal was used as the denominator to determine the signal ratio with other filters. Most signals measured with the Ag filter showed very low levels except for near the end of the discharge, as shown in Figure 8. Moreover, during the *I_p_* ramp-up phase, the signal level was even lower than the magnitude of the background noise fluctuation in the signal (~15 mV). This small signal created significant uncertainty in the signal ratio calculation. However, we could not further reduce the Ag thickness to enhance the signal level because it was already near the fabrication limit. As depicted in Figure 1a, the Ag filter has an energy cutoff around 100 eV. It was consequently inferred that the number of photons with energies above 100 eV was considerably low during the *I_p_* ramp-up phase. Therefore, another filter material was needed that has transmission lower than 100 eV. For this, we used a 1.0 μm Be filter, which has a slightly higher transmission energy than Al, as shown in Figure 1a.

**Figure 9:**
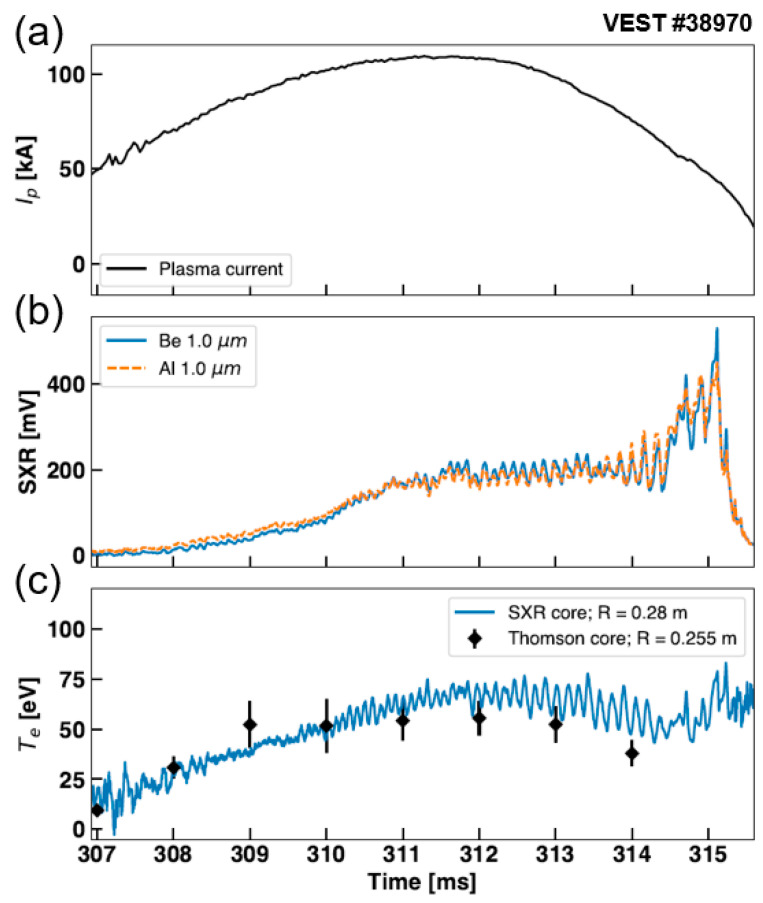
(**a**) Plasma current of discharge #38970. (**b**) Measured SXR signal using Al and Be filters, both taken from the same core-crossing LOS (R = 0.28 m at midplane). (**c**) Estimated *T_e_* using Be/Al ratio interpolation. The *T_e_* data from Thomson scattering (R = 0.255 m at midplane) are also plotted.

Figure 9 presents the results using a combination of Al and Be filters during Ohmic discharge. Figure 9a exhibits the plasma current of the discharge up to 110 kA. The signal level from the 1.0 μm Be filter on a channel passing through the plasma core was nearly identical to that from the Al filter also from the same LOS, as shown in Figure 9b. Furthermore, the timing of oscillations observed in both filter signals coincided. These oscillations persisted even in the signal ratio calculations, indicating that both signals were accurately measured from a nearly identical poloidal cross section. A closer examination of Figure 9b revealed that the Al filter signal was larger than the Be filter signal during the initial *I_p_* ramp-up phase (~310 ms). However, as the discharge proceeded, it was observed that the Be filter signal gradually increased, eventually surpassing the Al filter signal. This reversal in signal level was attributed to the Be transmission characteristics in the higher energy range compared to Al. From this observation, it could be inferred that *T_e_* increased gradually as *I_p_* increased.

To estimate *T_e_*, the signal ratio was interpolated onto the *R*(*T_e_*) curve of Be/Al. Figure 9c plots the time series of *T_e_* by interpolation. For comparison, Thomson scattering data obtained from the same discharge are also shown in the figure. We note that this is not an apples-to-apples comparison, since the Thomson data were locally measured at R = 0.255 m on the midplane (Z = 0 m), while the SXR data were from line-integrated measurements obtained from a core channel passing through R = 0.28 m on the midplane. Due to the mixing of signals from various plasma regions along the channel, a ratio of LOS signals cannot be directly compared with a local *T_e_* estimation. However, since SXR intensity is proportional to the square of electron density, SXR is mostly generated in the plasma core. Thus, the values estimated using the LOS ratio indirectly reflect the *T_e_* of the plasma core along the sight line [21]. Therefore, in this paper, a comparison of signal trends and order was made using SXR data from a region similar to the Thomson position. 

As shown in Figure 9b, it is noticeable that the SXR signal level was low during the early *I_p_* ramp-up period (307–310 ms), exhibiting a considerable uncertainty in the estimated values in Figure 9c. Nevertheless, the SXR *T_e_* continued to rise, consistent with the trend observed in the Thomson scattering measurements during the ramp-up phase. Beyond 312 ms, as *I_p_* decreased, *T_e_* started to decrease gradually. The decreasing *T_e_* was also observed in the Thomson data around a similar time point. While it was not an exact local measurement, the SXR *T_e_* demonstrated a fairly comparable trend with the Thomson *T_e_*, and the estimated values were within a similar order considering the error bars of the Thomson data. These results support the validity of the SXR-based *T_e_* measurements performed in this study. Furthermore, SXR measurements were advantageous in showing the temporal evolution of *T_e_* throughout the discharge. This stands as a clear advantage compared to Thomson measurements, which were taken at intervals of 1 ms. 

Rigorous local *T_e_* measurements using the two-filter method require the calculation of local emissivity through tomographic inversion, followed by the determination of local signal ratios. Exact local *T_e_* measurements will be conducted in the future using an additional two-filter SXR array, which is in preparation.

### 4.2. Consideration of Error Contribution from Impurity Line Emissions

In the previous section, we assumed that the measured SXR signals were emitted from a bremsstrahlung continuum. However, the characteristic lines that radiate from the impurities in the plasma could introduce errors in estimations through the two-filter method. In the case of VEST, residual gas analysis and spectroscopy results suggest that carbon and oxygen, apart from the hydrogen main fuel, could be considered as the main impurity species. To assess the impact of impurity line emissions on the calculated *R(T_e_)* curve used in the two-filter method, X-ray spectra from specific elements in the plasma were simulated using the FLYCHK code [22]. This calculation was performed under the assumption of the typical experimental condition in VEST after boronization, which is 1% composition of carbon and oxygen along with a plasma density of 10^19^ m^−3^ [23]. When we simulated X-ray spectra from plasma using a steady-state ionization balance [24], the transport and effective residence time of the impurity species were neglected. If the effects of transport and effective residence time are not included, all ions in the plasma are assumed to achieve their equilibrium charge state instantaneously. However, in real experimental conditions, ions may not have enough time to reach equilibrium due to their finite residence time by transport in the plasma. In this case, it is possible to overestimate the presence of highly ionized states, e.g., fully stripped ion state, which requires longer residence time compared to single or He-like ionized states. A previous study [25] also pointed out that the steady-state ionization balance model overestimated the presence of highly ionized states compared to the time-dependent model considering impurity transport. Therefore, the analysis based on steady-state ionization balance performed in this study can lead to more highly ionized impurities than are most likely to exist in VEST, resulting in overestimating the impurity effects on the *T_e_* measurements.

**Figure 10:**
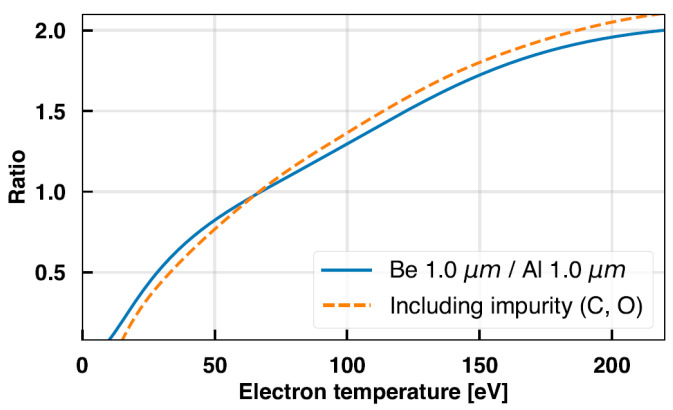
Ratio–temperature curves from the Be/Al filter set. The refined curve in consideration of impurities is shown as the dashed line.

Subsequently, the simulated X-ray spectra were used to derive a corrected *R(T_e_)* curve by considering filter transmission efficiency. Figure 10 plots the *R(T_e_)* curve from the Be/Al filter set obtained by supposing continuum radiation only along with a corrected curve including impurity effects. The calculation indicated an error of up to approximately 8% when carbon and oxygen impurities were included, consistent with the previous studies on the influence of low-Z impurities on the two-filter method [25,26,27]. It is noteworthy that Be and Al have an overlap in transmission range and similar transmission efficiencies, as shown in Figure 1a, suggesting that errors would be minimized when considering impurity characteristic lines in the case of the Be/Al combination used in this study. Based on the analysis results in this section and the comparison with Thomson data in the previous section, we can draw a conclusion that the two-filter method is applicable even in the presence of impurity contents by properly selecting the filter combination. In future work, we will use visual bremsstrahlung diagnostics currently in development to quantitatively identify and measure the concentration of impurities in order to further assess their error contribution.

## 5. Conclusions

A two-filter soft X-ray array has been developed for VEST to measure *T_e_* evolution with fast temporal resolution. The choice of filters was made considering the estimated VEST electron temperature (10–200 eV). We designed a pinhole camera consisting of two photodiodes facing different filters. We also used a filter wheel to find optimum filter parameters such as pinhole size, thickness, and filter material. The SXR array utilized a customized preamplifier, which was designed for low-noise applications. The measured signals showed sufficient signal levels for intensity ratio comparison. Experimental measurements made during VEST Ohmic discharges confirmed that the signals were not contaminated by stray light. The SXR array could also measure fluctuations similar to those detected by the Mirnov coil. An initial *T_e_* estimation was attempted using Be/Al filter signals, which showed sufficient signal levels throughout the discharge, including the current ramp-up phase. The estimated *T_e_* showed similar trends and signal levels to those from Thomson measurements, supporting the validity of the estimated *T_e_* by SXR array shown in this study. The effect of impurity line emissions on the estimation was also considered, where it was found that the estimated error contribution was not large (~8%) in typical VEST operation conditions. Through this study, we identified a new filter combination, Be/Al, suitable for a multichannel two-filter array, which can be utilized for low-*T_e_* (10–200 eV) measurements. The new filter combination suggested in this study will find applicability in other low-*T_e_* devices, contributing to expanding our measurement capabilities in low-temperature plasmas. Furthermore, the validity of this new approach is supported by comparing the results of the two-filter method with Thomson scattering data, which have not been presented in previous works on low-*T_e_* measurements based on the two-filter method.

In the future, another SXR array will be installed at a VEST mid-port to cover the whole plasma in a poloidal plane. With the addition of this array, the whole system will have 32 LOSs in a poloidal plane. Then, tomographic reconstruction will be applied to the multichannel data to obtain local *T_e_* profiles and two-dimensional (2D) *T_e_* evolution. The reconstructed 2D *T_e_* information will be important to study thermal transport in various VEST operation scenarios. In addition, impurity contributions to the two-filter estimation will be further investigated following impurity studies in VEST.

## Data Availability

Not applicable.
